# New insights into Blimp-1 in T lymphocytes: a divergent regulator of cell destiny and effector function

**DOI:** 10.1186/s12929-017-0354-8

**Published:** 2017-07-21

**Authors:** Shin-Huei Fu, Li-Tzu Yeh, Chin-Chen Chu, B. Lin-Ju Yen, Huey-Kang Sytwu

**Affiliations:** 1 Department and Graduate Institute of Microbiology and Immunology, National Defense Medical Center, 161, Section 6, Min-Chuan East Road, Neihu District, Taipei, 11490 Taiwan; 2 Department of Anesthesiology, Chi Mei Medical Center, Tainan, 71104 Taiwan; 3 Department of Recreation and Health-Care Management, Chia Nan University of Pharmacy and Science, Tainan, 71104 Taiwan; 4 Institute of Cellular and System Medicine, National Health Research Institutes, Zhunan, 35053 Taiwan

**Keywords:** Blimp-1, Prdm1, Bcl-6, CD4^+^ T lymphocytes, CD8^+^ T lymphocytes, T helper cells, Regulatory T cells, T follicular helper cells, Interleukins, STATs

## Abstract

B lymphocyte-induced maturation protein-1 (Blimp-1) serves as a master regulator of the development and function of antibody-producing B cells. Given that its function in T lymphocytes has been identified within the past decade, we review recent findings with emphasis on its role in coordinated control of gene expression during the development, differentiation, and function of T cells. Expression of Blimp-1 is mainly confined to activated T cells and is essential for the production of interleukin (IL)-10 by a subset of forkhead box (Fox)p3^+^ regulatory T cells with an effector phenotype. Blimp-1 is also required to induce cell elimination in the thymus and critically modulates peripheral T cell activation and proliferation. In addition, Blimp-1 promotes T helper (Th) 2 lineage commitment and limits Th1, Th17 and follicular helper T cell differentiation. Furthermore, Blimp-1 coordinates with other transcription factors to regulate expression of IL-2, IL-21 and IL-10 in effector T lymphocytes. In CD8^+^ T cells, Blimp-1 expression is distinct in heterogeneous populations at the stages of clonal expansion, differentiation, contraction and memory formation when they encounter antigens. Moreover, Blimp-1 plays a fundamental role in coordinating cytokine receptor signaling networks and transcriptional programs to regulate diverse aspects of the formation and function of effector and memory CD8^+^ T cells and their exhaustion. Blimp-1 also functions as a gatekeeper of T cell activation and suppression to prevent or dampen autoimmune disease, antiviral responses and antitumor immunity. In this review, we discuss the emerging roles of Blimp-1 in the complex regulation of gene networks that regulate the destiny and effector function of T cells and provide a Blimp-1-dominated transcriptional framework for T lymphocyte homeostasis.

## Background

B lymphocyte-induced maturation protein-1 (Blimp-1)^1^, a zinc-finger motif-containing transcriptional repressor, is encoded by the positive regulatory domain 1 gene (*Prdm1*)^1^ and was initially characterized as a negative regulator of β-interferon (IFN-β) gene expression [1]. Blimp-1 was further identified as a master regulator that orchestrates plasma cell development and the differentiation of immunoglobulin-secreting B lymphocytes [2, 3] and also controls the differentiation of the myeloid lineage [4]. The expression of Blimp-1 is dynamic in primordial germ cells and is critical for mouse embryonic development [5–7]. Generation of loss-of-function Blimp-1 mutants by gene targeting is embryonic-lethal in mice [8]. Therefore, Blimp-1 instructs diverse cell fates in the embryo and plays essential roles in multiple hematopoietic lineages.

The number of studies demonstrating the importance of Blimp-1 expression in different subsets of T lymphocytes for the regulation of immune networks has grown dramatically over recent years. Blimp-1 has been revealed as a key regulator of T cell homeostasis, and its ablation in T cells is responsible for downregulating expression of interleukin (IL)-10 and upregulating expression of IL-2 and gamma interferon (IFN-γ) [9, 10]. Recent experiments have demonstrated that Blimp-1 is also critical for CD4^+^ T helper (Th) cell differentiation. In CD4^+^ T cells, Blimp-1 inhibits Th1 differentiation [11] and opposes the formation of follicular helper T (Tfh) cells [12]. In contrast to the inhibition of Tfh commitment by constitutive expression of Blimp-1 in CD4^+^ T cells, deletion of Blimp-1 in CD4^+^ T cells augments Tfh differentiation [13]. Blimp-1 and interferon regulatory factor 4 (IRF4) were shown to be indispensable and to cooperate to regulate the expression of IL-10 and C-C chemokine receptor 6 (CCR6) in effector regulatory T (Treg) cells [14]. Furthermore, Blimp-1 instructs transcriptional regulation to control the expression of IL-2, IL-21 and IL-10 in effector CD4^+^ T cells for the maintenance of T cell homeostasis [14–19]. In addition to CD4^+^ T cells, Blimp-1 is also a critical component of the transcriptional program controlling the generation of heterogeneous CD8^+^ T cell populations. The importance of Blimp-1 in the formation of killer-cell lectin-like receptor G1 (KLRG1)^hi^IL-7 receptor (IL-7R)^lo^ short-lived effector cells (SLECs), KLRG1^lo^IL-7R^hi^ memory precursor effector cells (MPECs), effector memory (EM, KLRG1^hi^IL-7Rα^hi^CD62L^low^CCR7^low^) cells, central memory (CM, KLRG1^low^IL-7Rα^hi^CD62L^hi^CCR7^hi^) cells, and exhaustion of CD8^+^ T cells during immune responses has been demonstrated [20]. Moreover, Blimp-1 plays a critical role in the functions of CD8^+^ T cells including migration, cytotoxicity, survival, proliferation and cytokine production [20–22]. The genes regulated by the B cell lymphoma-6 (Bcl-6)/Blimp-1 axis serve as a cardinal switch to enable cytokine secretion and effector function predominantly in CD4^+^ and CD8^+^ T lymphocytes [23]. These studies highlight the complexity of the transcriptional programs coordinated by Blimp-1 for the development, differentiation and effector function of T lymphocytes. Here, we briefly review the findings concerning the significance of Blimp-1 in T lymphocytes and demonstrate divergent roles for Blimp-1 in different T lymphocyte lineages.

## The expression of Blimp-1 in T cell lineages

Blimp-1 is expressed not only in the B cell lineage but also in other cell lineages including T cells, granulocytes, macrophages, epithelial cells, retinal neurons, muscle cells and primordial germ cells [24]. In mice, the expression of Blimp-1 is detected in both CD4^+^ and CD8^+^ T cells that have the characteristics of effector and memory cells [9, 10].

### CD4^+^ T cells

Martins et al. reported that they could detect little steady-state expression of Blimp-1 mRNA in thymocytes by reverse transcription–quantitative polymerase chain reaction. Double-negative (DN) thymocytes, CD4 single-positive thymocytes and peripheral naïve CD4^+^ T cells expressed similar levels of Blimp-1 mRNA, which were threefold higher than the levels of Blimp-1 mRNA in double-positive (DP) thymocytes [10]. These results were similar to the results of another group who demonstrated Blimp-1 expression in DP thymocytes using microarray [25]. However, Kallies et al. did not detect any intrathymic Blimp-1 expression using a green fluorescent protein (GFP) knock-in strategy [9]. Martins et al. found that expression of Blimp-1 was higher in the memory, effector and regulatory T cell populations, and was induced after in vitro activation of naïve CD4^+^ T cells by T cell receptor (TCR) and/or IL-2 stimulation. The level of Blimp-1 mRNA in T cells was similar to that in lipopolysaccharide (LPS)-activated splenic plasma cells 6 days after in vitro stimulation with anti-CD3 and anti-CD28 antibodies and IL-2 [10]. Likewise, Kallies et al. demonstrated that the GFP^+^CD4^+^ T cells were CD44^hi^ and mainly CD62L^lo^, a cell surface phenotype indicating effector and memory CD4^+^ T cells, and that these cells showed high expression of other activation markers such as CD122 and glucocorticoid-induced tumor necrosis family related gene (GITR) protein [9]. Consistent with this, Gong et al. reported that Blimp-1 protein was expressed in both CD4^+^ and CD8^+^ T cells after anti-CD3 stimulation and that Blimp-1 protein levels in activated T cells were similar to those found in LPS-activated B cells by western blot analysis. Moreover, Blimp-1 expression in both CD4^+^ and CD8^+^ T cells was detected 24 h after activation with anti-CD3 antibody, and it was clearly expressed after 48 h in culture [15]. Taken together, these findings suggest that Blimp-1 expression is mainly confined to activated T cells.

Kallies et al. assessed Blimp-1 expression during the differentiation of GFP knock-in mouse CD4^+^ T cells into effector cells; they cultured CD62L^+^CD4^+^GFP^−^ T cells under Th1- or Th2-polarizing conditions. GFP analysis showed that Blimp-1 was induced in effector cells of both Th1 and Th2 lineages [9]. Salehi et al. sorted naive CD4^+^ T cells from Blimp-1-yellow fluorescent protein reporter mice and stimulated these cells under neutral, Th1, Th2 or Th17 conditions to analyze Blimp-1 mRNA expression at different time points. They found that cells cultured in Th1 or Th2 conditions began to express Blimp-1 sooner than cells cultured in neutral conditions but that Th1 cells expressed significantly more Blimp-1 than Th2 cells and at earlier time points. In contrast, cells cultured under Th17 conditions did not significantly upregulate Blimp-1, and only 5% of the cells expressed Blimp-1 at day 7.5 poststimulation. The differential expression of Blimp-1 during the differentiation of different Th populations was confirmed by either mRNA or protein analysis. Consistent with these findings, Blimp-1 was reported to be induced in Th1 and Th2 cells but not repressed in Th17 cells by transforming growth factor (TGF)-β [26]. Moreover, Blimp-1 mRNA was also detected in Treg cells [9, 10]. Cretney et al. examined the expression of Blimp-1 in Treg cells using GFP reporter mice and demonstrated that Blimp-1 was expressed in the subset of Foxp3^+^ Treg cells with an effector phenotype that produce IL-10. It was dispensable for the formation of effector Treg cells but essential for their ability to produce IL-10 [14].

### CD8^+^ T cells

#### Blimp-1 expression in different subsets of CD8^+^ T cells in mice

Previous studies demonstrated that in vitro stimulation with anti-CD3ε, anti-CD28 and IL-2 induced high levels of Blimp-1 mRNA expression in naïve T cells with delayed postactivation kinetics [9, 10], suggesting that the expression of Blimp-1 is enhanced in CD8^+^ T cells when they encounter a cognate antigen. Indeed, the amount of Blimp-1 was significantly increased in antigen-specific effector CD8^+^ T cells during acute influenza virus, lymphocytic choriomeningitis virus (LCMV) or vaccinia virus infection [27–29]. When they encounter antigen, naïve CD8^+^ T cells undergo differentiation to generate KLRG1^hi^IL-7R^lo^ SLECs and KLRG1^lo^IL-7R^hi^ MPECs that have different fates and potentials for memory cell development. After an infection is cleared, the MPECs will generate memory CD8^+^ T cells that can be either EM (KLRG1^hi^IL-7Rα^hi^CD62L^low^CCR7^low^) or CM (KLRG1^low^IL-7Rα^hi^CD62L^hi^CCR7^hi^) cells [22]. During the acute phase of LCMV infection, the IL-7Rα^low^ effector CD8^+^ T cells with high KLRG1 and low CCR7 mRNA expression exhibited elevated expression of Blimp-1 mRNA [30]. Blimp-1 expression was always higher in KLRG1^hi^IL-7R^lo^ SLECs than in KLRG1^lo^IL-7R^hi^ MPECs and remained heightened in CM T subsets after LCMV infection [28]. In contrast to the situation during acute viral infections, during chronic viral infections, virus-specific CD8^+^ T cells undergo an altered profile of transcription and become exhausted. Blimp-1 expression was higher in virus-specific CD8^+^ T cells undergoing exhaustion during chronic viral infection than in antigen-specific T cells after acute infection, suggesting a correlation between Blimp-1 expression and exhaustion [31]. Overall, these results indicate that during virus infection, Blimp-1 expression exhibits a heterogeneous pattern in different CD8^+^ T cell subsets.

#### The expression of BLIMP-1 in human CD8^+^ T cells

In addition to mouse T cells, the expression of BLIMP-1 in human CD8^+^ T cells was also demonstrated in several recent studies. In CD161^++^IL-18Rα^+^CD8^+^ human T cells, a newly identified subset of memory cells, the transcription level of BLIMP-1 was significantly higher than that in classical CD27^+^CD45RA^−^ memory CD8^+^ T cells. This high level of BLIMP-1 expression may contribute to the differentiated effector-type features of CD161^++^IL-18Rα^+^ CD8^+^ human T cells [32]. Lee et al. identified a novel population of IL-6Rα^hi^CD45RA^+/−^CCR7^−^CD8^+^ EM T cells, which may serve as a reservoir of effector CD8^+^ T cells. These IL-6Rα^hi^ CD8^+^ EM T cells produce high levels of Th2 cytokines and GATA binding protein 3 (GATA3), and are expanded in the peripheral blood mononuclear cells of asthma patients. Moreover, they express low levels of the transcription factors T-BET, Eomesodermin (EOMES) and BLIMP-1, suggesting that they are not terminally differentiated CD8^+^ T cells [33]. In addition to different expression levels in CD8^+^ T cell subsets, HIV-1 transactivator of transcription (Tat) protein treatment enhanced the transcription of *PRDM1* after T cell receptor stimulation. This effect of Tat on *PRDM1* expression was inhibited by blocking integrins, indicating that Tat modulates BLIMP-1 through the interaction of integrins with their ligands [34].

## The effects of Blimp-1 on T cell functions

Deletion of Blimp-1 in T cells leads to the dysregulation of T lymphocytes and the expression of an abnormally activated phenotype. This phenomenon is supported by evidence that Blimp-1 is necessary for normal thymocyte survival and controls T cell homeostasis. Blimp-1 is also critical for T helper differentiation and cytokine production.

### CD4^+^ T cells

#### Blimp-1 is important for thymocyte development

Martins et al. observed that the numbers of immature DP thymocytes are reduced and that they are prone to apoptosis in mice with T cell-specific Blimp-1 deletion generated using the proximal-*Lck*-Cre deletion system, suggesting that Blimp-1 may function in early T cell maturation and that its dysfunction is responsible for survival defects in DP thymocytes [10]. In addition, Lin et al. reported that Blimp-1 modulates lymphocyte development. The number of thymocytes in 6-week-old Blimp-1 transgenic mice was increased compared with controls and was even higher in conditional knockout (CKO) mice lacking Blimp-1 in T cells. The numbers of DP thymocytes in both transgenic and CKO mice were significantly increased compared with controls, suggesting that Blimp-1 may have a complicated and stage-dependent modulatory effect on thymocyte survival and/or expansion. After stimulation through the TCR or with phorbol-12-myristate-13-acetate plus ionomycin, the proliferation of thymocytes was significantly impaired in Blimp-1-transgenic mice compared with controls. In contrast, it was dramatically enhanced in Blimp-1-CKO mice compared with controls, indicating a suppressive role of Blimp-1 in thymocyte proliferation [35]. Deletion of Blimp-1 under control of *Cd4* or the proximal-*Lck* promoter resulted in global T cell defects during early thymic development. However, Blimp-1-deficient mice created using a distal-*Lck*-Cre system, which promotes deletion of genes during the late single-positive thymic development stage, had a normal number of thymocytes and did not show any signs of spontaneous autoimmunity [36]. Furthermore, Bach et al. showed that T cell-specific expression of the IL-2-inducible kinase-spleen tyrosine kinase (*Itk-Syk*) oncogene in mice leads to an early onset and aggressive polyclonal T cell lymphoproliferation. They found that high *Itk-Syk* expression in thymocytes induced Blimp-1-mediated premature terminal differentiation, resulting in oncogene-expressing cells being eliminated early in development [37]. Thus, Blimp-1 is required to induce cell elimination in the thymus.

#### Blimp-1 maintains peripheral homeostasis

Kallies et al. and Martins et al. both reported that Blimp-1 is expressed in effector and memory T cells. Kallies et al. generated Blimp-1-GFP knock-in mice and demonstrated that the GFP^+^ CD4^+^ T cells were effector and memory CD4^+^ T cells with high expression of activation markers such as CD122 and GITR, which accumulated in vivo and contributed to severe early-onset colitis [9]. Martins et al. showed that mice lacking Blimp-1 specifically in the T cell lineage had more effector CD4^+^ and CD8^+^ cells in the periphery [10]. Both mice with a T cell-specific deletion and Rag1^−/−^ mice reconstituted with *Prdm1* ^gfp/gfp^ fetal liver cells displayed a dysregulated population expansion that resulted in either T cell-mediated immune pathology or multiorgan infiltration, suggesting a linkage between Blimp-1 and the cell-intrinsic control of T cell activation and homeostasis [9, 10]. Studies from Sytwu’s group illustrated that Blimp-1 deficiency in T cells leads to higher numbers of activated CD4^+^ T cells, and this is associated with a homeostatic dysregulation of effector/memory T cells that contributes to both severe colitis in nonobese diabetic (NOD) mice and the exacerbation of autoimmune encephalomyelitis in myelin oligodendrocyte glycoprotein (MOG)_35–55_-immunized mice [35, 38, 39]. Therefore, Blimp-1 is a key regulator of effector T cells and controls their homeostasis.

Kallies et al. reported that Blimp-1 controls T cell proliferation and apoptosis. Blimp-1-mutant T cells are less susceptible to apoptosis than wild-type cells: when cell death was impaired in *Prdm1* ^gfp/gfp^ mice, the numbers of Blimp-1-mutant T cells increased, suggesting a mechanism that contributes to effector T cell expansion in vivo. They suggested that Blimp-1 in late-stage T cells controls activation-induced cell death (AICD) [9]. In contrast, Martins et al. showed that CKO and control CD4^+^ T cells were similarly susceptible to AICD. They demonstrated that when naive CKO CD4^+^ T cells were stimulated via the TCR, more cells produced IL-2 and proliferated than in wild-type mice [10]. Lin et al. demonstrated the inhibitory function of Blimp-1 on T cell proliferation in Blimp-1-transgenic mice. This downregulated proliferation may be the result of Blimp-1-mediated suppression of IL-2 production, because the production of IL-2 by stimulated transgenic CD4^+^ T cells was significantly decreased compared with that by control cells. In contrast, IL-2 production was remarkably increased in CKO T cells, indicating that Blimp-1 critically modulates T cell activation and proliferation [35]. Blimp-1-deficiency in T cells results in both enhanced proliferation and attenuated AICD, resulting in aberrantly large numbers of activated T cells. However, the detailed mechanism by which Blimp-1 regulates proliferation and cell death needs further investigation.

During chronic and acute viral infections, the antiviral T cell response is controlled through a host-regulated process. Hua et al. identified T-bet- and Blimp-1-dependent development of CD4^+^ T cells with cytotoxic potential and showed that this development was induced during influenza virus infection by antiviral type I IFNs and IL-2. Blimp-1 deficiency impaired the binding of T-bet to the *Gzmb* and *Prf1* promoters, suggesting that Blimp-1 controls the development of CD4^+^ T cells with cytotoxic potential by regulating the binding of T-bet to the promoters of the genes for cytolytic molecules [40]. In addition, increasing expression of IL-10 regulates the suppression of viral-specific T cell responses. A recent study demonstrated that virus-specific Th1 cells with elevated and sustained Blimp-1-dependent IL-10 expression displayed reduced inflammatory function during chronic LCMV infection [41]. Another study showed that Blimp-1 is highly expressed in CD4^+^ memory T cells compared with naive CD4^+^ T cells and that it limits HIV-1 transcription in CD4^+^ memory T cell subsets, the primary reservoir of latent HIV-1 [42]. Therefore, Blimp-1 plays an important role in regulating the effector function of CD4^+^ T cells during viral infections to maintain T cell homeostasis.

#### Blimp-1 controls T cell differentiations

Naïve CD4^+^ T cells can differentiate into different effector lineages including Th1, Th2, Th17 and Treg cells that express lineage-specific transcription factors (such as T-bet, GATA3, retinoic acid-related orphan receptor (ROR)γt or Foxp3) upon environmental stimulation and in a specific cytokine milieu [43]. Using a GFP knock-in strategy to delete Blimp-1 in T cells, it was demonstrated that *Prdm1* ^gfp/gfp^ CD4^+^ T cells can differentiate into Th1 and Th2 effector cells that secrete levels of IFN-γ, IL-4 and IL-10 similar to those produced by wild-type effector cells, indicating that Blimp-1 is not required for initiation of differentiation and cytokine production. These findings suggest that early CD4^+^ T cell effector differentiation is independent of Blimp-1 and that Blimp-1 expression is not essential for the acquisition of effector functions in the T-cell lineage [9]. Blimp-1 controls the differentiation of some Th cell lineages, including promoting Th2 lineage commitment by opposing the differentiation of IFN-γ-secreting Th1 cells [11], antagonizing follicular Th (Tfh) cells [12] and cooperating with IRF4 to maintain the function of Treg cells [14]. Th1 cells also express Blimp-1 to limit Tfh lineage commitment by suppressing the expression of both Bcl6 and C-X-C chemokine receptor type 5 (CXCR5), a chemokine receptor that is characterized as a signature marker for Tfh cells migrating into B-cell follicles [44, 45]. Recently, several studies reported that Blimp-1 regulates IL-17-secreting Th17 cells [26, 35, 39]. Lin et al. demonstrated that transgenic expression of Blimp-1 in T cells attenuates autoimmune diabetes through suppression of Th17 cells, while Blimp-1 deficiency leads to an increase in Th17 cells [35]. In addition, Blimp-1 regulates and maintains homeostasis of the intestinal mucosa by limiting the numbers of Th17 cells [26]. Disruption of the IL-23–Th17 axis ameliorates the severity of T cell-specific Blimp-1 deficiency-mediated colitis in CKO mice [39]. Elevated susceptibility to experimental autoimmune encephalomyelitis in Blimp-1-deficient mice involves increased Th1 and Th17 responses [38]. Deletion of Blimp-1 in T cells leads to the inability to suppress Th1 and Th17 cells in mice with colitis or autoimmune diabetes [9, 10, 26, 35, 39]. Therefore, Blimp-1 is clearly central to effector CD4^+^ T cell differentiation.

#### The interplay between Blimp-1 and cytokines

Kallies et al. and Martins et al. agree that Blimp-1-deficient CD4^+^ T cells produce higher levels of IL-2 and IFN-γ but less IL-10 and IL-4 [9, 10] than wild-type cells. Further reports showed that the mean fluorescence intensity of IFN-γ staining per cell in these CKO CD4^+^ T cells was increased, indicating that each CKO cell produces more IFN-γ. Blimp-1 attenuates IFN-γ production in CD4^+^ T cells activated in vitro under nonpolarizing conditions and in vivo [11, 35, 38]. Wang et al. showed that Blimp-1 is very strongly induced and plays a role in IL-2 inhibition when naive T cells are stimulated in the presence of IL-4, both in vitro and in vivo [46]. IL-2 is critical for T cell immunity to promote proliferation, activation and differentiation of T cells [47]; it induces Blimp-1 expression in activated T cells and inhibits its own production through the induction of Blimp-1 in a negative feedback loop [15, 16]. Lack of Blimp-1 expression in CD4^+^ cells under the control of the proximal*-Lck* or *Cd4* promoters leads to intrinsic functional defects and an increase in IL-17-producing cells in vivo, establishing a new role for Blimp-1 in regulating IL-17 production [26, 35, 38, 39]. The overexpansion of Th1 and Th17 cells in CKO mice was significantly reduced by introducing a Blimp-1 transgene, supporting the crucial role of Blimp-1 in autoimmunity [35, 38]. Thymic deletion of Blimp-1 in T cells results in T cell development defects and spontaneous autoimmunity. However, peripheral deletion of Blimp-1 driven by the distal-*Lck* promoter led to reduced Th17 activation and reduced severity of autoimmune encephalomyelitis. Jain et al. also identified Blimp-1 as a key transcription factor induced by IL-23 to drive the inflammatory function of Th17 cells by enhancing expression of IL-23 receptor, granulocyte-macrophage colony stimulating factor and IFN-γ in the peripheral T cells [36].

IL-21 is a pleiotropic cytokine that induces expression of Blimp-1 that is controlled by cooperation between signal transducer and activator of transcription 3 (STAT3) and IRF4 [17]. A high percentage and absolute number of IL-21-producing CD4^+^ T cells were observed in MOG_35–55_-immunized Blimp-1-deficient mice, and the numbers of central nervous system (CNS)-infiltrating Th1, Th17, IFN-γ^+^IL-17A^+^ and IL-21^+^IL-17A^+^ CD4^+^ T cells were markedly increased in the brain and spinal cord of these Blimp-1 CKO mice at an early effector phase, suggesting a critical role of Blimp-1 in control of IL-21 production [38]. These findings raise the possibility that a negative feedback loop exists wherein IL-21 inhibits its own production through induction of Blimp-1; this possibility needs to be further investigated.

IL-10 is an anti-inflammatory cytokine produced by CD4^+^ T cells, Treg cells, CD8^+^ T cells, dendritic cells (DCs), macrophages and B cells [48]. IL-10-producing CD4^+^ T cells have been reported to be a self-regulation mechanism during viral or parasitic infections [49, 50]. A population of effector T cells producing IL-10 (IL-10^+^IFN-γ^+^ double producers) defined as T regulatory 1 (Tr1) cells is responsible for T-cell plasticity or reprogramming [51]. Blimp-1 has been implicated as a key transcription factor involved in the molecular mechanisms directing IL-10 production in effector T cells. Blimp-1-deficient CD4^+^ T cells produce less IL-10 [9, 10], and targeting tumor necrosis factor receptor 1 assembly in Blimp-1 CKO mice regulates Th1/Th17 effector status by increasing the frequency of IL-10-producing cells and the levels of IL-10 in Th1 and Th17 cells [39]. Virus-specific T cells self-limit their responsiveness and reduce their inflammatory function via Blimp-1-dependent IL-10 expression during chronic LCMV infection [41]. Type I interferon-mediated induction of Blimp-1, and a subsequent expansion of Tr1 cells, has been reported to limit *Plasmodium*-specific Tfh accumulation and to constrain antimalarial humoral immunity during blood-stage *Plasmodium* infection [52]. In addition, IL-27, together with TGF-β, is critical for IL-10 production in Th1-driven immune responses both in vitro and in models of infection with *Toxoplasma gondii* [53, 54]. Moreover, IL-23 counteracts the IL-27- and IL-12-mediated effects on Blimp-1-induced Tr1 development and stabilizes the inflammatory Th17 phenotype, leading to uncontrolled Th17 cell-driven CNS pathology [18]. TGF-β antagonizes Blimp-1, is a key driver of IL-10 production in proinflammatory effector T cells downstream of IL-12 and IL-27, and shifts IL-10 regulation from a Blimp-1-dependent to a Blimp-1-independent pathway by inducing c-Maf in Tr1 cells [19]. Importantly, Blimp-1-dependent IL-10 production by Tr1 cells is a major regulator of tumor necrosis factor (TNF)-mediated inflammation [55]. In summary, these studies demonstrate an essential role for Blimp-1 in the transcriptional framework regulating the intrinsic plasticity of Th cells in an inflammatory milieu.

#### Blimp-1 controls regulatory T cell function

Treg cells are required for peripheral tolerance, and Blimp-1 is a target of Foxp3 in Treg cells [56]. Because Treg cells are dependent on IL-2 for their maintenance, the feedback regulatory loop between Blimp-1 and IL-2 shown in activated T cells may also be important for Treg homeostasis.

IL-10 production by Tregs is significantly downregulated in Blimp-1-CKO mice, suggesting that Blimp-1 has a critical role in Treg function, which is important for limiting severe T cell-mediated immune pathology [10, 38]. However, Kallies et al. reported that Blimp-1-deficient Treg cells protect lymphopenic hosts from colitis elicited by injection of T cell populations depleted of Treg cells [9]. Intriguingly, Blimp-1 overexpression upregulates the suppressive ability of Treg cells in Blimp-1-transgenic or Blimp-1-CKO mice, suggesting that Blimp-1 critically modulates and rescues the expansion and functions of Tregs [35, 38]. Importantly, the acquisition of Treg effector functions in Foxp3^+^ Tregs by production of IL-10 also requires the expression of Blimp-1 [14, 57]. A population of follicular regulatory T (T_FR_) cells expressing Blimp-1was identified in the germinal center, which limited Tfh cell and germinal center B cell numbers. Notably, Bcl-6 is essential for T_FR_ cell formation, and Blimp-1 limits the numbers of T_FR_ cells, suggesting that Bcl-6 coordinates with Blimp-1 to control T_FR_ formation and homeostasis [58]. Blimp-1 was also upregulated in association with the activation of virus-reactive T-bet^+^ Treg and with acquired expression of IL-10 in a mouse model of influenza virus infection, thereby conferring a functional specialization to an antiviral immune response [59]. Furthermore, Blimp-1, together with elevated levels of TGF-β, IL-10, IFN-β and CXCR3, plays a crucial role in the ability of graft-infiltrating Foxp3^+^ Treg cells to maintain spontaneously induced kidney allograft tolerance in the DBA/2 (H-2^d^) to C57BL/6 (H-2^b^) mouse strain combination [60]. Therefore, upregulation of Blimp-1 is essential for modulating the immunoregulatory and effector functions of Treg cells.

#### Blimp-1 is associated with CD4^+^ T cell exhaustion

During chronic viral infection, both the CD4^+^ and CD8^+^ T cell responses are impaired by a dysfunctional or exhausted state characterized by diminished effector function and enhanced expression of inhibitory molecules in T cells [61]. Higher levels of BLIMP-1 are expressed in T cells from patients with progressive chronic HIV infection [62] and are associated with lower levels of HIV expression in memory CD4^+^ T cells from nonprogressors [63]. BLIMP-1 is also induced in T cells stimulated by HIV-pulsed DCs and is associated at both the RNA and protein levels with other protein markers of exhaustion, including programmed death-1 (PD-1), lymphocyte activation gene-3 (LAG-3), cytolytic T-lymphocyte antigen-4 (CTLA-4) and T-cell immunoglobulin mucin-containing domain-3 (TIM-3) [64, 65]. Interestingly, expression of BLIMP-1 is translationally regulated by microRNA miR-9 [66]. Reduced levels of miR-9 in CD4^+^ T cells have been shown to play a functional role in the higher levels of BLIMP-1 expression in patients with progressive chronic HIV infection who have reduced IL-2 expression and generalized T-cell dysfunction, indicating a novel miR-9/BLIMP-1/IL-2 axis that is dysregulated in progressive HIV infection [62].

Elevated expression of IL-10 mediated by Blimp-1 is involved in the suppression of viral-specific T cell responses during the course of chronic LCMV infection [41]. In addition, Blimp-1 is a critical regulator of CD4 T cell exhaustion with elevated levels of inhibitory factors being expressed during chronic toxoplasmosis [67]. Therefore, Blimp-1 is highly upregulated in exhausted CD4^+^ T cells.

### CD8^+^ T cells

#### The effects of Blimp-1 on CD8^+^ T cell differentiation

Over recent years, the functions of Blimp-1 in programming the differentiation of CD8^+^ T cells have been gradually established. During acute LCMV infection, a deficiency of Blimp-1 in activated CD8^+^ T cells (*Prdm1* ^*flox/flox*^ GzB-cre^+^ mice) disturbs the normal expression of several cytolytic molecules. Blimp-1-deficient CD8^+^ T cells acquired mature memory features including enhanced survival, proliferation potential, IL-2 production and increased formation of KLRG1^lo^IL-7R^hi^ MPECs as well as CD62L^hi^ CM CD8^+^ T cells at early time points after infection. In addition, in the absence of Blimp-1, the transition rate from effector to memory cells was increased, indicating that Blimp-1 is critical in the development of CD8^+^ T cells during viral infection at stages from terminal differentiation to memory cell maturation [28]. During chronic infection, virus-specific CD8^+^ T cells become exhausted and accompanied by a hierarchical loss of effector function and sustained expression of several inhibitory molecules. In exhausted CD8^+^ T cells, Blimp-1 plays an important role in the regulation of expression of inhibitory molecules including PD-1, 2B4, LAG-3 and CD160, indicating that it has a role in controlling T cell exhaustion during chronic viral infection. Moreover, Blimp-1 functions as a transcriptional rheostat that intrinsically regulates the effector function and the exhaustion of CD8^+^ T cells at low and high expression levels, respectively [31]. In addition to its role during viral infection, Blimp-1 also affects effector CD8^+^ T cell differentiation during DC vaccination. However, the expansion of CD8^+^ T cells and the formation of functional memory T cells is not affected in Blimp-1-deficient OT-I cells responding to DC vaccination, suggesting a critical role for Blimp-1 in the formation of SLECs but not MPECs in the absence of inflammation [68].

IRF4 directly binds to the regulatory elements of *Prdm1* to control Blimp-1 expression. During *Listeria monocytogenes* infection, *Irf4* ^*−/−*^ CD8^+^ T cells retained a “precursor-like” state with impaired acquisition of an effector phenotype that was similar to that of *Prdm1* ^*−/−*^ CD8^+^ T cells, suggesting that IRF-4 functions upstream of Blimp-1 in the development of protective effector CD8^+^ T cells during an immune response against an intracellular bacterium [69]. During virus infection, signals via costimulatory molecules and cytokines are critical for the generation of effector CD8^+^ T cells. After virus clearance, downregulation of costimulatory and cytokine receptors may promote apoptosis of the effector population. Blimp-1 can recruit the histone-modifying enzymes G9a and histone deacetylase 2 (HDAC2) to the regulatory elements of *Il2ra* and *Cd27*, thereby repressing the expression of these genes, further dictating the fate of effector CD8^+^ T cells [70]. Previous studies established a critical role for Blimp-1 in integrating inflammation and antigen signaling during effector T cell priming. Recently, Stelekati et al. demonstrated that the negative impact of persistent LCMV infection on CD127^+^KLRG1^−^ memory CD8^+^ T cell development was abolished in *Prdm1* ^flox/flox^ *Gzmb*-Cre OT1 cells, suggesting that Blimp-1 regulates memory CD8^+^ T cell differentiation in the presence of bystander chronic infection and prolonged inflammation [71].

#### The effects of Blimp-1 on effector functions of CD8^+^ T cells

Blimp-1, Bcl-6, T-bet and Eomes orchestrate a transcriptional program that regulates the differentiation of effector and memory CD8^+^ T cells. In addition to cell differentiation, Blimp-1 is required for the function of cytotoxic T cells. Kallies et al. demonstrated that Blimp-1 is required for the migration of viral antigen-specific CD8^+^ T cells from lymph nodes into the lungs during influenza virus infection. They observed that Blimp-1-deficient T cells had decreased and elevated expression of lung-homing CCR5 and lymph organ-localizing CCR7, respectively, suggesting that Blimp-1 suppresses CCR7 expression to control the efficient trafficking of CD8^+^ T cells from lymph nodes to peripheral tissues [27]. Blimp-1 is also required for the cytotoxic function of CD8^+^ T cells. Conditional deletion of Blimp-1 in activated CD8^+^ T cells (*Prdm1* ^flox/flox^ *Gzmb*-Cre) did not affect the production of effector cytokines and CD107a but attenuated the granzyme B expression and cytotoxicity of viral antigen-specific CD8^+^ T cells during chronic infection [31]. Rutishauser et al. also observed that Blimp-1-deficient cytotoxic T cells had decreased granzyme B expression after acute LCMV infection. Moreover, their results revealed that the percentage of polyfunctional (IFN-γ, TNF-α and IL-2 triple cytokine-producing) cells was increased in *Prdm1* ^*−/−*^ (*Prdm1* ^*flox/flox*^ *GzB-Cre*) mice. In addition to altering effector molecule expression, Blimp-1 antagonized the proliferation of virus-specific effector CD8^+^ T cells stimulated by viral antigens and homeostatic cytokines [28].

Inhibitor of DNA binding 3 (Id3) is expressed by effector CD8^+^ T cells and supports their survival during the effector-to-memory cell transition. Ji et al. demonstrated that Blimp-1 triggers the death of terminally differentiated CD8^+^ T cells through directly repressing Id3 expression and consequently increasing E2A transcriptional activity [29]. It is well established that CD25, a subunit of the IL-2 receptor, and CD27, a costimulatory molecule in the TNF receptor family, play important roles in regulating CD8^+^ responses, proliferation and survival during the different stages of viral infection [72–75]. Blimp-1 acts as an epigenetic regulator to control the chromatin state of *Cd25* and *Cd27* by recruiting histone-modifying enzymes G9a and HDAC2, but not Ezh2, in CD8^+^ T cells at the peak of the response to LCMV infection, suggesting that Blimp-1 downregulates cytokine receptor expression to promote the death of effector cells [70].

In contrast to its function as a repressor, Blimp-1 can also function as an enhancer of IL-10 production. During influenza virus infection, antiviral CD8^+^ cytotoxic T lymphocytes produce IL-10 to prevent excess inflammation [76]. Sun et al. demonstrated that CD4^+^ T cell-produced IL-2 and innate cell-derived IL-27 act synergistically via Blimp-1 to amplify IL-10 production in CD8^+^ T cells in the respiratory tract during influenza virus infection [77]. Moreover, the authors recently demonstrated that type I interferons can enhance the synergistic effect of IL-2 and IL-27 to promote Blimp-1-mediatedI L-10 production by effector CD8^+^ T cells during influenza infection [78].

## Transcriptional regulation of Blimp-1 in T cells

Blimp-1 in T cells is induced upon activation. Blimp-1 is associated with an abundance of chromatin-modifying enzymes that induce epigenetic changes at specific targets or recruit corepressor complexes to mediate gene silencing to regulate diverse cell fates [24].

### CD4^+^ T cells

#### Transcriptional control of Blimp-1 in CD4^+^ T cells

It has been reported that nuclear factor-κB signaling is required for induction of *Prdm1* expression in B cells [79]. In addition, the BTB and CNC homology 2 (Bach2) protein functions mainly to repress Blimp-1 in B cells [4]. Recently, Bach2 was shown to be expressed also in T cells and to function as a critical regulator suppressing EM-related genes in naive T cells [80]. The expression of Bach2 mRNA is high in CD4 single-positive thymocytes, Foxp3^+^ CD4 single-positive thymocytes, naïve T cells, splenic CD4^+^ and CD8^+^ T cells, and Treg cells in the spleen, but very low in DN, DP and CD8 single-positive thymocytes [80, 81]. Although the protein expression of Bach2 in naive T cells is high, it is lower than that in B cells [80]. Bach2 plays crucial roles in CD4^+^ T cell differentiation, generation of EM T cells and survival and development of Treg cells by regulating the effector and differentiation transcriptional program. Blimp-1, upregulated in EM T cells, is repressed by Bach2 in T cells, consistent with its repression by Bach2 in B cells [80–82]. The level of *Prdm1* expression was elevated in Bach2^−/−^ Treg cells or naive Bach2^−/−^ CD4 T cells after TCR stimulation [80, 82]. Binding of Bach2 to *Prdm1* was measured in induced Treg cells by chromatin immunoprecipitation with massively parallel sequencing (ChIP-Seq) [81]. Bach2 protein functions mainly to repress Blimp-1 in T cells to regulate T cell homeostasis, activation and differentiation. Other studies have also indicated that the abundance of Blimp-1, and consequently the secretion of proinflammatory cytokines, is regulated by enhancing miR-9 expression to target the 3′ untranslated region of *Prdm1* upon TCR activation [62, 66]. Together, these data indicate that Blimp-1 can be regulated at transcriptional and posttranscriptional levels.

#### Transcriptional involvement of Blimp-1 in effector T cells

Blimp-1 is expressed in memory and effector populations of T cells [9, 10]. Blimp-1 antagonizes the expression of Bcl-6 to regulate the effector function and differentiation program not only of B cells but also of T cells [23]. Martins et al. reported that Blimp-1 CKO CD4^+^ effector T cells had twice the abundance of *Bcl6* mRNA transcripts as did control effector cells, indicating that Bcl-6 repression was impaired in Blimp-1-deleted CD4^+^ effector T cells [10]. Moreover, Bach2 suppresses the EM-related expression of ST-2, Blimp-1, IL-10 and S100a to maintain the naïve status of T cells in a cell-intrinsic manner. Expression of these EM-related proteins was upregulated in Bach2^−/−^ naive T cells [80]. Therefore, Blimp-1 is a critical component in the complex genetic programs that control effector and memory lymphocytes.

#### Cooperation of Blimp-1 with transcription factors in T helper differentiation

An emerging role of Blimp-1 is to regulate differentiation programs in T cells. The Bcl-6 and Blimp-1 regulatory axis is critical for B cell differentiation, while Blimp-1 expression is repressed by Bcl-6 in mature B cells. In T cells, Blimp-1 also functions as an antagonistic transcription factor because Blimp-1 expression is suppressed when Bcl-6 expression is initiated [10]. Initial reports have indicated high levels of Bcl-6 expression in the Tfh cells responsible for the antigen-specific regulation of B cell immunity, while high levels of Blimp-1 are expressed in non-Tfh cells [12, 23, 83].

Bcl-6 is involved in Th1 differentiation by repressing Th2 cytokine expression via decreasing GATA3 protein levels [84] and repressing IL-5 transcription [85]. In contrast to Bcl-6, Blimp-1 counteracts Th1 differentiation during Th2 lineage commitment by directly binding to *Ifng*, *Tbx21* and *Bcl6* genes. Blimp-1 mRNA and protein are more highly expressed in Th2 cells than in Th1 cells, and mice lacking Blimp-1 in CD4^+^ T cells exhibit impaired humoral Th2 responses. However, Bcl-6 mRNA is more highly expressed in Th1 cells than in Th2 cells [11]. In addition, Bach2^−/−^ naive T cells have increased expression of IL-4, IL-10, Blimp-1 and GATA3, suggesting that a lack of the Blimp-1 repressor in T cells predisposes them to differentiate into Th2 cells [80]. The suppressive effect of Blimp-1 in Th1 cells is supported by evidence that transgenic Blimp-1 expression in T cells attenuates Th1 cell expansion through downregulation of *Tbx21* and *Ifng* [35]. Other studies also indicated that Blimp-1 is able to bind to at least one site in the *Il17a* gene in Th2 cells but that this is not sufficient to downregulate *Il17a* transcription in cells stimulated under Th17 conditions [26]. Blimp-1 is reported to impede the development of Th17 cells via *Rora* and *Rorc* downregulation after transgenic Blimp-1 expression in Blimp-1 deficient T cells under control of the proximal*-Lck* promoter [35]. However, peripheral deletion of Blimp-1 resulted in reduced Th17 activation, and IL-23-induced Blimp-1 was found to colocalize with RORγt, STAT3 and p300 at the *Il23r*, *Il17a/f* and *Csf2* cytokine genes to enhance their expression and to drive the inflammatory function of Th17 cells [36]. The mechanism by which Blimp-1 regulates Th17-mediated immunopathology depends on the model that is used, and it causes either thymic T cell developmental defects or deletion of genes during the late SP thymocyte developmental stage that continues in peripheral T cells.

Bcl-6 orchestrates Tfh cell lineage commitment to support B cell maturation into antibody-producing cells [86], whereas Blimp-1 functions as an antagonistic transcription factor to oppose Tfh cell differentiation [12]. Fazilleau et al. demonstrated that the differentiation and diversity of effector Tfh cells in vivo was related to the strength of TCR binding. Expression of Blimp-1 distinguished “lymphoid” Th effector cells (CD62L^hi^CCR7^hi^) from those Bcl-6-expressing CXCR5^hi^ “resident” effector Tfh cells (CD62L^lo^CCR7^lo^) by high expression of IL-4, IL-21 and PD-1 after stimulation with TCR of higher affinity [83]. Multiple signals are involved in negatively regulating Tfh cells. Recent studies have reported that STAT5 signaling, induced by IL-2, negatively regulates Tfh cell differentiation and controls humoral immunity and B cell tolerance by upregulating Blimp-1 to repress Bcl6 expression, suggesting that the IL-2/STAT5 axis functions to regulate Blimp-1 expression [87, 88]. It has been reported that there is a flexibility between Th1 and Tfh-like gene expression patterns as a result of strong IL-2 signaling that decreases the ratio of Bcl-6 to T-bet and controls the Bcl-6–Blimp-1 axis, leading to Blimp-1-mediated repression of Tfh signature genes in effector Th1 cells [45]. Another transcription factor, Kruppel-like factor 2 (KLF2), binds to the promoter region of *Prdm1* and restricts Tfh cell differentiation by inducing Blimp-1 to inhibit Bcl-6 expression after T cell activation [89]. Notably, recent studies have demonstrated a critical role for T cell factor 1 (TCF-1) function upstream of the Bcl-6–Blimp-1 axis to direct the differentiation of the Tfh lineage [90–92]. After viral infection, effector CD4^+^ T cells differentiate into TCF-1^high^Blimp-1^low^ Tfh and TCF-1^low^Blimp-1^high^ Th1 cells. In the absence of TCF-1, cells were unable to maintain the transcriptional and metabolic signatures of Tfh cells and displayed an abnormal “Th1-like” gene expression profile with increased expression of *Il2ra* and *Prdm1*, which limit the Tfh response [91]. TCF-1 was also found to bind directly to the *Bcl6* promoter and *Prdm1* 5′ regulatory regions, resulting in activation of Bcl-6 but repression of Blimp-1 [92]. Downregulation of TCF-1 binding to the *Prdm1* intron leads to upregulation of Blimp-1 and Blimp-1-mediated repression of Bcl-6 in Th1 cells, while its retention on the upstream region of *Bcl6* in Tfh cells results in upregulation of Bcl-6 and suppression of Blimp-1 during Tfh differentiation [90]. Consequently, the balance between Bcl-6 and Blimp-1 expression in T cells plays an essential role in regulating T cell differentiation.

#### Transcriptional regulation of Blimp-1 in cytokine production

Blimp-1 is also crucial for inducing cytokine production by inflammatory T helper cells and effector Treg cells.

The gene encoding IL-2 is one of the most important genes targeted by Blimp-1 in T cells, because IL-2 production is indispensable for T cell proliferation and differentiation. The relationship between IL-2 and Blimp-1 was reported as a negative feedback loop in which IL-2 signaling induces *Prdm1* transcription and Blimp-1 represses *Il2* transcription in T cells [15]. Further studies reveal that IL-2 production in Blimp-1-deficient CD4^+^ T cells is upregulated upon TCR stimulation and that Blimp-1 in T cells represses IL-2 production by direct repression of *Il2* and *Fos* transcription [10, 16]. Furthermore, IL-21-activated STAT3 is a potent inducer of Blimp-1 expression in B cells [93] and CD4^+^ T cells [17]. The molecular basis for IL-21-mediated Blimp-1 induction in CD4^+^ T cells was clarified by the identification of an IL-21 response element downstream of *Prdm1* that binds STAT3 and IRF4, which cooperatively mediate signaling and are required for optimal *Prdm1* expression [17].

IRF4 regulates the activation of Blimp-1 expression not only during plasma cell differentiation [94] but also in all effector Treg cells by binding strongly to two previously identified binding sites in the 3′ region and between exons 5 and 6 of *Prdm1* (conserved noncoding sequence 9) [14]. Strong binding of IRF4 to the first introns of *Il10* and *Ccr6* and binding of Blimp-1 specifically to intron 1 of the *Il10* locus were further identified by chromatin immunoprecipitation (ChIP) analysis, suggesting that IRF4 together with Blimp-1 regulates *Il10* expression in Treg cells. This study also demonstrated that both IRF4 and Blimp-1 are required for active histone modification and that the IRF4–Blimp-1 axis is essential for the acquisition of Treg cell effector functions [14]. Consistent with these features of effector Treg cells, T_FR_ cells express elevated levels of Blimp-1, IL-10, GITR, CTLA-4 and inducible T cell costimulator [58]. Blimp-1 expression specifies a distinct population of effector Treg cells expressing the anti-inflammatory cytokine IL-10 and is important for the function and homeostasis of Treg cells.

Blimp-1 deficiency in T cells results in downregulation of IL-10 production [9, 10], and Blimp-1 is critical for IL-10 expression in Treg cells [14]. Likewise, the IL-27-mediated induction of IL-10 in CD8^+^ T cells depends on Blimp-1 [77]. An early study of IL-27 signal transduction for IL-10 production in CD4^+^ T cells indicated that the involvement of early growth response gene 2 (Egr-2) and Blimp-1 is required for IL-10 production in CD4^+^ T cells and controls the balance between regulatory and inflammatory cytokines. Furthermore, this study demonstrated that IL-27-induced expression of Egr-2, which binds to the promoter region of *Prdm1* to activate its transcription, is dependent on STAT3 in CD4^+^ T cells [95]. Published studies have further identified an essential function for Blimp-1 in IL-10 production induced by IL-27 in inflammatory T helper cells [18, 19]. Notably, precommitted Th17 cells adopt an IL-27- and IL-12-mediated Tr1-like phenotype, producing IL-10 and IFN-γ, by upregulating Blimp-1, while IL-12 signaling results in phosphorylation of STAT4, which binds directly to regulatory elements of *Prdm1* [18]. Blimp-1 is also essential for IL-10 expression by Th1 cells through direct binding to a regulatory element in the *Il10* locus that is mainly dependent on IL-12-mediated activation of STAT4, which binds to conserved noncoding sequencesin the *Prdm1* locus and to the same region (conserved noncoding sequence-9) as Blimp-1 in the *Il10* locus. In addition, c-Maf acts synergistically with Blimp-1 to induce IL-10 expression in Th1 cells by binding to the conserved noncoding sequence-9 region in the *Il10* promoter, the same region bound by Blimp-1 and STAT4. c-Maf further enhances Blimp-1 expression by binding to the intron 5 Maf recognition site but not to the promoter of *Prdm1*, suggesting that it interferes with the repressive function of Bach2 by binding to the same DNA motif [19]. These studies have also demonstrated that IL-27 induces Blimp-1-dependent IL-10 production in Th cells, whereas TGF-β antagonizes Blimp-1 expression and mediates IL-10 production driven by c-Maf and AhR [18, 19], consistent with a previous report that TGF-β acts as a suppressor of Blimp-1 expression during Th17 differentiation [26]. Therefore, Blimp-1 regulates cytokine production by T cells via a complex pathway coordinated by diverse transcriptional programs depending on various stimuli from the surrounding environment.

### CD8^+^ T cells

#### The molecular regulation of Blimp-1 expression in CD8^+^ T cells

The molecular regulation of Blimp-1 expression is distinct in naïve, effector and memory CD8^+^ T cells. Previous studies demonstrated that additional culture of activated CD8^+^ T cells in IL-2, IL-4 or IL-12 but not in IL-15 maintains the expression of Blimp-1 [15]. IL-21 induces higher and more rapid expression of *Prdm1* in T cells than does IL-4 stimulation. The induction of *Prdm1* expression by IL-21 was abrogated and diminished in *Stat3* ^*−/−*^ and *Irf4* ^*−/−*^ T cells, indicating that IL-21-mediated *Prdm1* gene expression is dependent on STAT3 and IRF4. ChIP and luciferase assay experiments revealed that STAT3 and IRF4 broadly cooperate to regulate IL-21-induced *Prdm1* gene expression in T cells [17, 69]. Cui et al. demonstrated that the IL-21–IL-10–STAT3 pathway is critical to the differentiation, maturation and self-renewal of memory CD8^+^ T cells during LCMV infection through regulating individual transcription factors including Blimp-1, Eomes and Bcl-6. The amounts of Eomes, T-bet, Bcl-6 and Blimp-1 protein in *Stat3* ^*−/−*^ CD8^+^ T cells are comparable to those in *Stat3* ^*+/+*^ cells at day 8 after LCMV infection, suggesting that STAT3 signaling is not critical to the translational expression of these molecules in the differentiation of effector CD8^+^ T cells. However, the expression of Blimp-1, Eomes and Bcl-6 was significantly decreased in *Stat3* ^*−/−*^ memory T cells compared with *Stat3* ^*+/+*^ memory cells, suggesting that IL-21–IL10–STAT3 signaling necessarily regulates Blimp-1 expression during the effector-to-memory transition [96].

During DC vaccination, the expression of Blimp-1 was correlated with the number of antigen-specific T cells. The expression of *Prdm1* was more highly induced in effectors when low numbers (10^4^) compared with high (10^6^) numbers of OT-I cells were transferred prior to DC vaccination. Moreover, the induction of *Prdm1* was dependent on IL-2, indicating that the IL-2/Blimp-1 axis is a key regulator of SLEC differentiation in vivo in this low-inflammation model of DC immunization [68]. During influenza virus infection, the splenic IL-2Rα-deficient antigen-specific CD8^+^ T cells fail to develop into KLRG1^+^IL-7R^−^ SLECs and express less Blimp-1 than wild-type cells. However, the differentiated *Il2Ra* ^*−/−*^ antigen-specific SLECs express high levels of Blimp-1, indicating that IL-2 signaling is not essential for Blimp-1 expression but is required for its optimal expression in CD8^+^ T cells during virus infection. Moreover, IL-2–STAT5 can cooperate with IL-12–STAT4 to induce high amounts of Blimp-1 and SLEC differentiation [97].

The Hippo pathway, a conserved developmental system triggered by cell–cell contact signals to trigger differentiation, induces yes-associated protein degradation and Blimp-1 expression [98]. Rodriguez et al. demonstrated that suppressor of cytokine signaling1 (*Socs1)* ^*−/−*^ MHC-I-restricted premelanosome protein-1 (Pmel-1) transgenic TCR CD8^+^ T cells expressed higher levels of Blimp-1 upon stimulation with cognate self-antigen (mgp10025–33) than did wild type Pmel-1 cells, suggesting that SOCS1 regulates Blimp-1. However, the underlying mechanism of this effect is unknown [99]. Kurachi et al. demonstrated that a basic leucine zipper transcription factor (BATF) is essential for operation of the differentiation checkpoint in early effector CD8^+^ T cells. BATF binds to regulatory regions in *Prdm1* and many other genes encoding effector transcription factors to form a “BATF-centric” interaction network of transcription factors to regulate the differentiation of effector CD8^+^ T cells [100]. Moreover, BATF overexpression enhances Blimp-1 and granzyme B expression to promote the quality and quantity of virus-specific CD8^+^ T cells during infection. In addition, the IL-21–STAT3–BATF axis cooperates with antigen-induced IRF4 to maintain Blimp-1 expression and CD8^+^ T cell effector functions [101].

Recently, Yamada et al. demonstrated that a deficiency of menin, a tumor suppressor protein, in CD8^+^ T cells will result in impaired immune responses of antigen-specific CD8^+^ T cells to infection. Their results revealed that menin inhibits terminal effector differentiation and enhances memory development by suppressing expression of T-bet and Blimp-1 [102]. Although the underlying mechanism by which menin suppresses Blimp-1 expression is unknown, the authors suggest that menin interacts with JunD and acts as a repressor of AP-1.

#### The molecular regulation of Blimp-1 in CD8^+^ T cell functions

Growing evidence suggests that the interactions between Blimp-1 and other factors mediate counter-regulatory influences to produce functional T cells. After an acute influenza virus infection, the transcriptional profiles of *Tbx21*, *Eomes* and *Bcl6* are changed in virus-specific Blimp-1-deficient CD8^+^ T cells, suggesting that Blimp-1 is required for the differentiation of effector CD8^+^ T cells by regulating the transcriptional programs of effector and memory T cell differentiation [27]. Recently, Xin et al. demonstrated that Blimp-1 cooperates with T-bet to drive effector CD8^+^ T cell differentiation by regulating overlapping and distinct transcriptional signatures during virus infection. T-bet overexpression partially compensates for KLRG1 expression and downregulates IL-7R and Eomes in Blimp-1-deficient CD8^+^ T cells during viral antigen-specific SLEC differentiation and memory cell formation. However, T-bet protein expression does not differ significantly between antigen-specific wild-type and Blimp-1-deficient CD8^+^ T cells during influenza virus infection, indicating that the expression of T-bet protein in CD8^+^ T cells is largely independent of Blimp-1 [97]. Id2 and Id3 are expressed by effector CD8^+^ T cells and support their survival during the naïve-to-effector cell and effector-to-memory cell transitions, respectively [103, 104]. Ji et al. demonstrated that Blimp-1 represses Id3 expression by directly targeting the *Id3* promoter in effector CD8^+^ T cells. Id3 regulates the survival of SLECs partly through antagonizing the binding of E2A to DNA, suggesting that the Blimp-1–Id3–E2A axis determines the fate of effector CD8^+^ T cells [29]. In addition to Id3, Blimp-1 directly regulates *Il2ra* and *Cd27* expression through recruitment of histone-modifying enzymes H3 methyltransferase G9a and HDAC2, indicating that Blimp-1 acts as an epigenetic regulator to regulate effector CD8^+^ T cell development in response to an acute virus challenge [70]. Moreover, Blimp-1 directly represses *Pd1* transcription by regulating expression of nuclear factor of activated T cells (NFAT)c1, altering local chromatin structure and evicting NFATc1 from its binding sites on the *Pd1* gene during the early stages of effector CD8^+^ T cell differentiation after acute virus infection [105].

## Genetic disruption of Blimp-1 in T cells and its effect on predisposition to disease

The role of Blimp-1 in autoimmune diseases, infectious diseases and lymphoid malignancies has been studied intensively.

### Animal disease models

Blimp-1 is expressed in effector T cells and is required for controlling their homeostasis. Mice either lacking Blimp-1 specifically in T cells or reconstituted with Blimp-1-deficient fetal liver cells develop progressive colitis or a lethal wasting disease with increased effector CD4^+^ and CD8^+^ T lymphocyte infiltration [9, 10]. C57BL/6 mice in which Blimp-1 is ablated develop severe colitis. A similar phenotype is observed in NOD mice with T cell-specific Blimp-1 disruption that have increased Th1/Th17 effector cell populations [35, 39], while transgenic Blimp-1 attenuates the diabetogenic effect of lymphocytes and thereby ameliorates the disease progression of autoimmune diabetes in NOD mice [35]. Blimp-1 is also able to suppress autoimmune encephalomyelitis through downregulation of Th1 and Th17 cells [38].

The function of Blimp-1 has been studied in multiple infectious disease models. During influenza virus infection, deficiency of Blimp-1 in T cells (*Prdm1* ^*flox/flox*^ proximal-*Lck-Cre*) will lead to a delayed recovery from infection and increased cellular infiltration in the lungs, indicating a significant role of Blimp-1 in T cell responses against influenza infection [27]. Although Blimp-1-deficient memory CD8^+^ T cells are capable of providing protection during a second LCMV infection [28], double mutant *Tbx21* ^*−/−*^ *Prdm1* ^*flox/flox*^ *Lck*-Cre mice showed accelerated weight loss and death during LCMV infection compared with single-mutant and wild-type mice, indicating that Blimp-1 cooperates with T-bet for the differentiation of protective effector CD8^+^ T cells [97].

Blimp-1 also participates in the development of T cell lymphoma. High expression of the *Itk-Syk* oncogene in thymocytes induces Blimp-1 expression regulated by STAT3 and IRF4 cooperation. Furthermore, the high *Itk-Syk*-expressing thymocytes may undergo Blimp-1-mediated premature terminal differentiation, leading to the elimination of oncogene-expressing cells at an early developmental stage. In contrast, the expression of Blimp-1 was not observed in lymphocytes expressing low levels of *Itk-Syk*. Therefore, low and high expression levels of the *Itk-Syk* fusion transcript induce early and delayed onset of clonal T cell lymphoma, respectively, through regulating Blimp-1 expression [37].

### Human diseases

BLIMP-1 is considered to be a candidate tumor suppressor gene in lymphoid malignancies. Early studies indicated that BLIMP-1β lacking its PR domain and having a diminished capacity to repress target genes was expressed in myeloma cell lines [106], and mutational inactivation of BLIMP-1 has been identified in a subset of diffuse large B-cell lymphomas [107, 108]. An involvement of BLIMP-1β in T cell lymphoma was also reported, where a high expression level was correlated with chemotherapy resistance [109]. Another study has also indicated that loss of BLIMP-1 occurs in anaplastic large T-cell lymphomas [110]. In addition, BLIMP-1 is inactivated in extranodal NK/T-cell lymphoma, nasal type (EN-NK/T-NT) where its downregulation is mediated by miR-223, providing a prognostic indicator for evaluating the clinical outcomes of EN-NK/T-NT patients [111]. Recent study reported that infiltration of BLIMP-1^+^ FOXP3^+^ effector Treg cells into tumor can improve prediction of disease recurrence in a cohort of colorectal cancer patients [112]. BLIMP-1 has been further identified as an important factor in T cell exhaustion during progressive chronic HIV infection. IL-2-induced expression of BLIMP-1 is repressed by upregulation of miR-9, which leads to reduced binding of BLIMP-1 to the *IL2* promoter. Published studies have further identified that a regulatory miR-9/Blimp-1/IL-2 pathway is impaired in progressive HIV disease [21, 62]. Low expression of *PRDM1* was associated with high HIV genome transcription levels in resting CD4^+^ CM T cells, suggesting that BLIMP-1 might be involved in controlling the HIV reservoirs in the CM T cell subset [63]. Therefore, BLIMP-1 functions as a gatekeeper of T cell activation and suppression to prevent or dampen autoimmune disease, antiviral responses and antitumor immunity.

## Conclusion

**Fig. 1:**
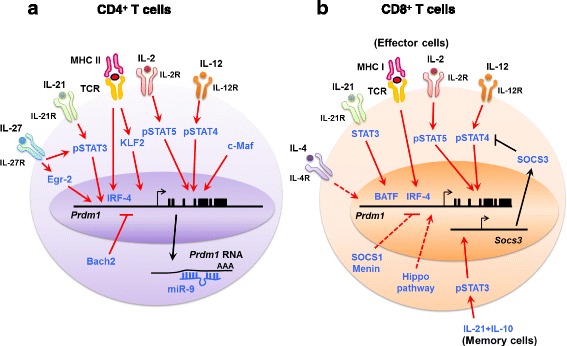
Regulators for Blimp-1 expression in **a** CD4^+^ T cells and **b** CD8^+^ T cells. Bach2, BTB and CNC homology 2; BATF, Basic leucine zipper transcription factor; Blimp-1, B lymphocyte-induced maturation protein-1; Egr-2, Early growth response gene 2; IL, Interleukin; IRF4, Interferon regulatory factor 4; KLF2, Kruppel-like factor 2; MHC II, Major histocompatibility complex class II; Prdm1, Positive regulatory domain 1; SOCS, Suppressor of cytokine signaling; STAT, Signal transducers and activators of transcription; TCR, T cell receptor. The solid line indicates direct action by activation of expression of the *Prdm1* gene. The dashed line indicates regulations that require further investigation for underlying mechanisms

**Fig. 2:**
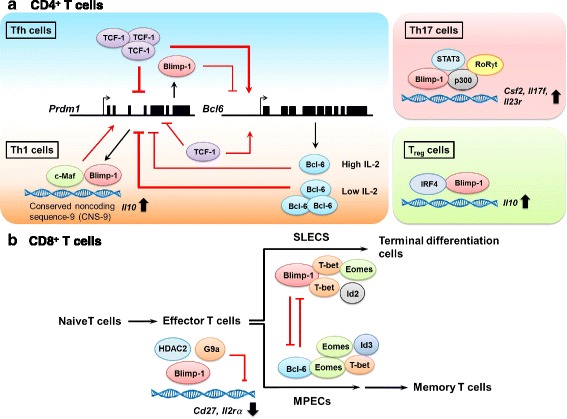
Blimp-1 cooperates with different molecules to regulate the differentiation and function of **a** CD4^+^ T cells and **b** CD8^+^ T cells. Bcl-6, B cell lymphoma-6; Blimp-1, B lymphocyte-induced maturation protein-1; Eomes, Eomesodermin; HDAC2, Histone deacetylase 2; Id2, Inhibitor of DNA binding 2; Id3, Inhibitor of DNA binding 3; IL, Interleukin; IRF4, Interferon regulatory factor 4; MPECs, Memory precursor effector cells; RORγt, Retinoic acid-related orphan receptor γt; SLECs, Short-lived effector cells; STAT, Signal transducers and activators of transcription; TCF-1, T cell factor 1; Tfh, Follicular helper T cells; Th, T helper

**Fig. 3:**
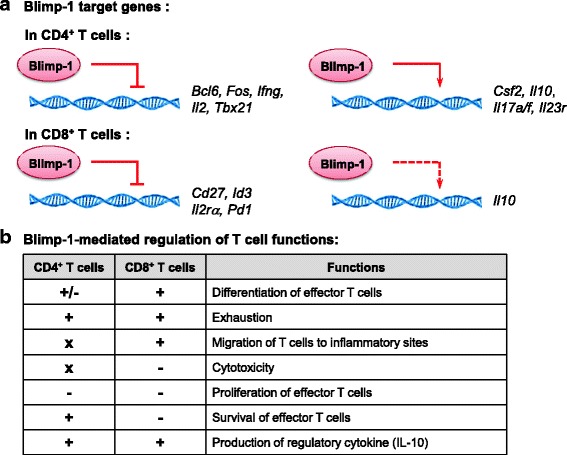
Overview of Blimp-1-mediated regulations in T cells. **a** Broad influence of Blimp-1 in the expression of different molecules. Bcl-6, B cell lymphoma-6; Blimp-1, B lymphocyte-induced maturation protein-1; Id3, Inhibitor of DNA binding 3; IFN-γ, interferon-γ; IL, Interleukin; IL2ra, Interleukin 2 receptor subunit alpha; PD-1, Programmed death-1. The solid line indicates direct action. The dashed line indicates regulations that require further investigation for underlying mechanisms. **b** Blimp-1 regulates divergent functions of T cells. +: Positive regulation; −: Negative regulation; x: Unidentified regulation

This review has focused on the findings over the past decade that have led to a better understanding of the essential role of Blimp-1 in instructing T cell destiny and effector functions. Expression of Blimp-1 is observed in both CD4^+^ and CD8^+^ T cells (Fig. 1), and its expression promotes the differentiation and cytokine production of effector T cells through cooperation with other transcription factors while suppressing the transcriptional signatures of naïve and memory T cells (Fig. 2). Blimp-1 is conventionally regarded as a repressor that regulates T cell differentiation and function, but notably, Blimp-1 is identified as an enhancer of IL-10 production to fine-tune the extent of inflammation and injury (Fig. 3). It is intriguing that Blimp-1 together with cooperating transcription factors can function as either an activator or a repressor and can determine the fate of multiple T-cell lineages. Understanding the expression patterns of transcriptional regulators in T cell subsets suggests that the determination of activation and repression of T cells is a combinatorial process mediated by these molecules to maintain immune homeostasis. Because Blimp-1 appears to orchestrate cascades of explicit gene expression programs in T lymphocytes, studying Blimp-1 and identifying its target genes has revealed important aspects of this regulatory machinery and may help to provide important insights into the regulation of immune homeostasis and the potential for therapeutic intervention.

## Abbreviations

AICD: Activation-induced cell death
Bach2: BTB and CNC homology 2
BATF: Basic leucine zipper transcription factor
Bcl-6: B cell lymphoma-6
Blimp-1: B lymphocyte-induced maturation protein-1
CCR6: C-C chemokine receptor 6
ChIP: Chromatin immunoprecipitation
ChIP-Seq: Chromatin immunoprecipitation with massively parallel sequencing
CKO: Conditional knockout
CM: Central memory
CNS: Central nervous system
CTLA-4: Cytolytic T-lymphocyte antigen-4
CXCR5: C-X-C chemokine receptor type 5
DCs: Dendritic cells
DN: Double-negative
DP: Double-positive
Egr-2: Early growth response gene 2
EM: Effector memory
EN-NK/T-NT: Extranodal NK/T-cell lymphoma, nasal type
Eomes: Eomesodermin
Foxp3: Forkhead box p3
GATA3: GATA binding protein 3
GFP: Green fluorescent protein
GITR: Glucocorticoid-induced tumor necrosis family related gene
HDAC2: Histone deacetylase 2
Id2: Inhibitor of DNA binding 2
Id3: Inhibitor of DNA binding 3
IFN-β: Interferon-β
IFN-γ: Interferon-γ
IL: Interleukin
IL2ra: Interleukin 2 receptor subunit alpha
IL-7R: IL-7 receptor
IRF4: Interferon regulatory factor 4
Itk-Syk: IL-2-inducible kinase-spleen tyrosine kinase
KLF2: Kruppel-like factor 2
KLRG1: Killer-cell lectin-like receptor G1
LAG-3: Lymphocyte activation gene-3
LCMV: Lymphocytic choriomeningitis virus
LPS: Lipopolysaccharide
MOG: Myelin oligodendrocyte glycoprotein
MPECs: Memory precursor effector cells
NFATc: Nuclear factor of activated T cells
NOD mice: Non-obese diabetic mice
PD-1: Programmed death-1
Pmel-1: Premelanosome protein-1
Prdm1: Positive regulatory domain 1
RORγt: Retinoic acid-related orphan receptor γt
SLECs: Short-lived effector cells
SOCS: Suppressor of cytokine signaling
STAT: Signal transducers and activators of transcription
Tat: Transactivator of transcription
TCF-1: T cell factor 1
TCR: T cell receptor
Tfh: Follicular helper T cells
T_FR_: Follicular regulatory T cells
TGF-β: Transforming growth factor-β
Th: T helper
TIM-3: T-cell immunoglobulin mucin-containing domain-3
TNF: Tumor necrosis factor
Tr1 cells: T regulatory 1 cells
Treg: Regulatory T cells

## Footnotes

1 Fully capitalized *PRDM1* and BLIMP-1 were referred as human gene and protein names, respectively, while only an initial capital letter of *Prdm1* and Blimp-1 were represented as mouse gene and protein names, respectively.
